# The Effect of Inflammatory Status on Butyrate and Folate Uptake by Tumoral (Caco-2) and Non-Tumoral (IEC-6) Intestinal Epithelial Cells

**DOI:** 10.22074/cellj.2017.4859

**Published:** 2017-05-17

**Authors:** Mafalda R. Couto, Pedro Gonçalves, Telmo A. Catarino, Fátima Martel

**Affiliations:** 1 Department of Biochemistry, Faculty of Medicine and Institute for Research and Innovation in Health Sciences, University of Porto, Porto, Portugal; 2 Innate Immunity Unit, Institute Pasteur, French National Institute of Health and Medical Research (INSERM), U668, Paris, France

**Keywords:** Tumor Necrosis Factor-α, Interferon-γ, Butyrate, Folic Acid, Colorectal Cancer

## Abstract

**Objective:**

Colorectal cancer (CRC) is the second leading cause of cancer death in occidental countries. Chronic inflammatory bowel disease (crohn’s disease and ulcerative colitis) is associated with an increased risk for CRC development. The aim of this work was to investigate the relationship between inflammatory status and absorption of nutrients with a role in CRC pathogenesis.

**Materials and Methods:**

In this experimental study, we evaluated the *in vitro* effect of tumour necrosis factor-alpha (TNF-α), interferon-γ (IF-γ), and acetylsalicylic acid on 14C-butyrate (14C- BT), 3H-folic acid (3H-FA) uptake, and on proliferation, viability and differentiation of Caco-2 and IEC-6 cells in culture.

**Results:**

The proinflammatory cytokines TNF-α and INF-γ were found to decrease uptake of a low concentration of 14C-BT (10 µM) by Caco-2 (tumoral) and IEC-6 (normal) intestinal epithelial cell lines. However, the effect of TNF-α and INF-γ in IEC-6 cells is most probably related to a cytotoxic and antiproliferative impact. In contrast, INF-γ increases uptake of a high concentration (10 mM) of 14C-BT in Caco-2 cells. The anticarcinogenic effect of BT (10 mM) in these cells is not affected by the presence of this cytokine. On the other hand, acetylsalicylic acid stimulates 14C-BT uptake by Caco-2 cells and potentiates its antiproliferative effect. Finally, both TNF-α and INF-γ cause a significant decrease in 3H-FA uptake by Caco-2 cells.

**Conclusion:**

The inflammatory status has an impact upon cellular uptake of BT and FA, two nutrients with a role in CRC pathogenesis. Moreover, the anti-inflammatory acetylsalicylic acid potentiates the anticarcinogenic effect of BT in Caco-2 cells by increasing its cellular uptake.

## Introduction

Colorectal cancer (CRC) is the second leading cause of cancer death in occidental countries (1). The causes of CRC are multifactorial. The risk factors for CRC include age, family history, environmental, and dietary factors (2). Epidemiological studies have highlighted the role of diet in CRC risk: a good correlation between a diet high in saturated fats and low in dietary fiber, fruit, and vegetables, and an increased risk of CRC development has been well established (3). Butyrate (BT), a product of intestinal microbiota fermentation of dietary fiber, has a protective role in the prevention and progression of colorectal carcinogenesis (4). Inhibition of colon carcinogenesis by BT involves epigenetic regulation of gene expression, related to histone deacetylase inhibition, causing hyperacetylation of histones (5, 6). Moreover, BT has other intracellular targets including DNA methylation, histone methylation, hyperacetylation of nonhistone proteins, selective inhibition of histone phosphorylation, regulation of the expression of micro-RNAs, (miRNA) and modulation of intracellular kinase signaling (1, 7). Given that the anticarcinogenic effect of BT depend on its intracellular concentration, the mechanisms involved in its cellular uptake at the intestinal epithelial level will have a major impact in its activity.

Folate is a vitamin B contained generally in fresh fruits and green vegetables and is essential for normal DNA synthesis and replication as well as for epigenetic regulation of gene expression (8, 9). Folate deficiency causes genomic instability, chromosomal breaks, and aberrant DNA methylation might lead to cancer (10). In previous epidemiologic and clinical studies, the risk of CRC was directly related to low levels of folate and multiple case-control. Observational cohort studies report a reduction in CRC risk for patients with high levels of folate consumption (3). However, animal studies have suggested that high levels of folate might promote colorectal oncogenesis by enhancing pre-existing adenomatous polyps (11). So, the protection provided by dietary folate supplementation might depend on the stage of colorectal carcinogenesis: folate may have a protective role against neoplasia in normal colorectal mucosa, but it may enhance pre-existing adenomatous lesions (3).

Chronic inflammatory bowel disease (IBD, Crohn’s disease and ulcerative colitis) is associated with an increased risk of CRC (12). The most common forms of IBD, Crohn’s disease, primarily affects the small intestine and colon and is associated with an exaggerated production of T helper type 1 (Th1) cytokines such as tumor necrosis factor-alpha (TNF-α), interferon-gamma (IFN-γ) and IL-18 (13). On the other hand, the regular use of nonsteroidal anti-inflammatory drugs (NSAIDs) such as aspirin (acetylsalicylic acid, ASA) has a protective effect on CRC development (14). The aim of this study was to further investigate the relationship between inflammatory status and absorption of nutrients with a role in CRC pathogenesis. More specifically, the effect of proinflammatory Th1 citokines and anti- inflammatory agent ASA on BT uptake by normal and tumoral intestinal epithelial was investigated and if these compounds affect the anticarcinogenic effect of BT. Moreover, we investigated the effect of proinflammatory Th1 cytokines upon folic acid uptake. 

## Materials and Methods

In this experimental study, we used: [^14^ C] BT ([1-^14^ C]-n-butyric acid sodium salt; specific activity 30-60 mCi/mmol) (Biotrend Chemikalien GmbH, Koln, Germany); acetic acid, dimethylsulfoxide (DMSO), bovine serum albumin, ethanol, N-2-hydroxyethylpiperazine- N0-2-ethanesulfonic acid (HEPES), INF-γ, (2-[N-morpholino] ethanesulfonic acid hydrate) (MES), Minimum Essential Medium, nicotinamide adenine dinucleotide (NADH), sodium pyruvate, p-nitrophenylphosphate, penicillin/streptomycin/amphotericin B solution, sulforhodamine B, Tris, trypsin-EDTA solution, TNF-α (Sigma, St. Louis, MO, USA); perchloric acid, triton X-100 (Merck, Germany). Compounds tested were dissolved in ethanol or PBS with 0.1% bovine serum albumin and added to the culture medium and buffer ( 1% (v/v)). 

### IEC-6 and Caco-2 cell culture

The IEC-6 and Caco-2 cell lines were obtained from the Deutsche Sammlung von Mikroorganismen und Zellkulturen (Braunschweig, Germany) and used between passages numbers 19-27 (IEC-6 cells) and 23-40 (Caco-2 cells). The cells were maintained in a humidified atmosphere of 5% CO_2_ /95% air. IEC-6 cells were cultured in Dulbecco’s Modified Eagle’s Medium: RPMI 1640 medium (1:1), supplemented with 10% fetal bovine serum, 0.1 U/ml insulin, 5.96 g HEPES, 2.2 g NaHCO_3_ , 100 U/ml penicillin, 100 mg/ml streptomycin and 0.25 mg/ml amphotericin B (all from Sigma, St. Louis, MI, USA). Caco-2 cells were cultured in Minimum Essential Medium containing 5.55 mM glucose, 15% fetal calf serum, 25 mM HEPES, 100 U/ml penicillin, 100 mg/ml streptomycin and 0.25 mg/ml amphotericin B (all from Sigma, St. Louis, MI, USA). Culture medium was changed every 2-3 days and the culture split every 7 days. For subculturing, the cells were treated with 0.25% trypsin-EDTA (5 minutes, 37˚C), split 1:3, and subcultured in plastic culture dishes (21-cm^2^, Corning Costar, Corning, NY, USA). For the experiments, cells were seeded on 24-well plastic cell culture clusters (1.9 cm^2^, TPPs, Trasadingen, Switzerland), and the experiments were performed 8-10 days after the initial seeding. 

### Chronic treatment of cells

Caco-2 or IEC-6 cells were treated for 24 hours with TNF-α (1-200 ng/ml), INF-γ (1-200 ng/ml), ASA (0.5 mM) or BT (10 mM) before transport, proliferation, viability, and differentiation studies. Control cells were exposed to the respective solvents. 

### Uptake studies

Transport experiments were performed with Caco-2 and IEC-6 cells incubated in glucose-free Krebs (GFK) buffer containing (in mM) 125 NaCl, 4.8 KCl, 1.2 MgSO_4_ , 1.2 CaCl_2_ , 25 NaHCO_3_ , 1.6 KH_2_ PO_4_ , 0.4 K_2_ HPO_4_ , and 20 MES, pH=6.5 (^14^C-BT experiments) or pH=5.5 (^3^H-FA experiments). After the removal of the culture medium, the cells were washed twice with 0.3 ml of GFK buffer at 37˚C. Then, 0.3 ml of GFK buffer containing 14 C-BT (10 µM or 10 mM) or ^3^H-FA (10 nM) at 37˚C was added. Incubation was stopped after 3 minutes (^14^C-BT experiments) or 6 minutes (^3^H-FA experiments) by removing the incubation medium, placing the cells on ice, and rinsing the cells with 0.5 ml of ice-cold GFK buffer. The cells were then solubilized with 0.3 ml of 0.1% (v/v) Triton X-100 (in 5 mM Tris HCl, pH=7.4) and radioactivity was measured by liquid scintillation counting after overnight at room temperature. Drugs to be tested were present during the incubation period. 

### Determination of cellular proliferation (sulforhodamine B assay)

After the treatment period (24 hours), 62.5 µl of ice-cold 50% (wt/vol) trichloroacetic acid (TCA) were added to the culture medium (500 µl) on each well to fix cells (1 hour at 4˚C in the dark). After being washed with tap water five times, plates were air-dried and then stained for 15 minutes with 0.4% (wt/vol) sulforhodamine B (SRB) dissolved in 1% (vol/vol) acetic acid. SRB was removed, and cultures rinsed four times with 1% (vol/vol) acetic acid to remove residual dye and dried. Then, the bound dye was solubilized with 375 µl of 10 mM Tris•NaOH solution (pH=10.5). The absorbance of each well was determined at 540 nm. Then samples diluted to obtain absorbance values lower than 0.7. 

### Quantification of cellular viability (lactate dehydrogenase assay)

After the treatment period (24 hours), cellular leakage of the cytosolic enzyme lactate dehydrogenase (LDH) into the extracellular (culture) medium was spectrophotometrically measured by quantification of the decrease in absorbance of NADH during the reduction of pyruvate to lactate, as described (15). 

### Determination of cellular differentiation (alkaline phosphatase activity assay)

After the treatment period (24 hours), cell differentiation was measured by quantification of alkaline phosphatase (ALP) activity, as previously described (16). ALP activity was determined spectrophotometrically by using p-nitrophenylphosphate as substrate, and the results were expressed as nmol p-nitrophenol minute^-1^ mg protein^-1^ . 

### Protein determination

The protein content of cell monolayers was determined as described (17), using human serum albumin as standard. 

### Statistical analysis

Arithmetic means are given with SEM. N indicates the number of replicates of at least three different experiments. Statistical significance of the difference between two groups was evaluated by the Student’s t test; statistical analysis of the difference between various groups evaluated by the ANOVA test, followed by the Student- Newman-Keuls test. Differences were considered to be significant when P<0.05. 

## Results

### Effect of proinflammatory cytokines and ASA on ^14^C-BT uptake by IEC-6 and Caco-2 cells 

**Fig.1:**
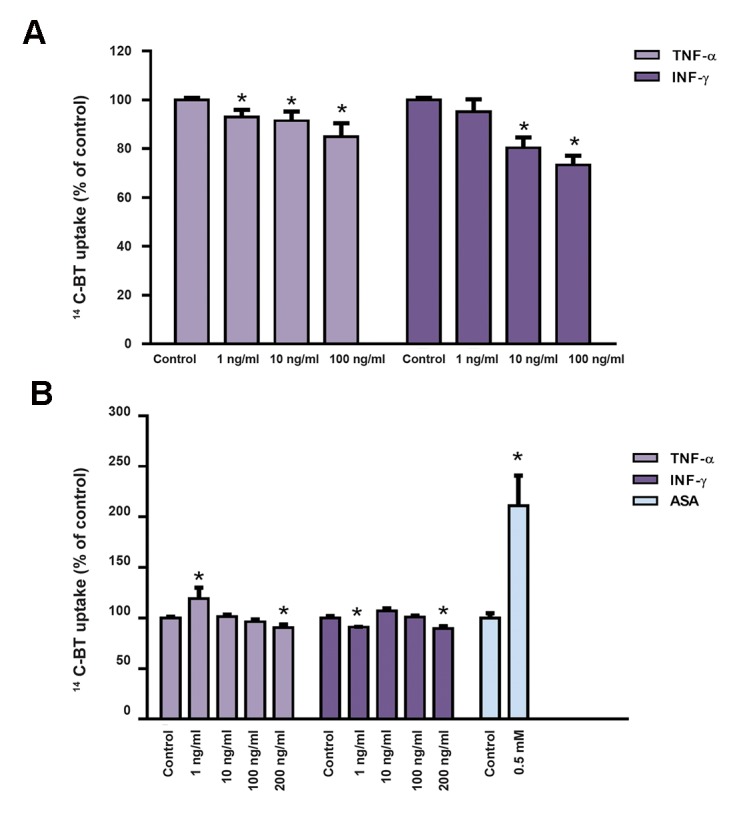
Effect of TNF-α (n=3-21), INF-γ (n=3-18) and ASA (n=16) (24 hours) on the uptake of a low concentration (10 µM) of ^14^C-BTby A. IEC-6 and B. Caco-2 cells. Results are presented as arithmetic means ± SEM. TNF-α; TNF-tumour necrosis factor-alpha, INF-γ; Interferon-γ, ASA; Acetylsalicylic acid, and *; Significantly different from control (P<0.05).

**Fig.2:**
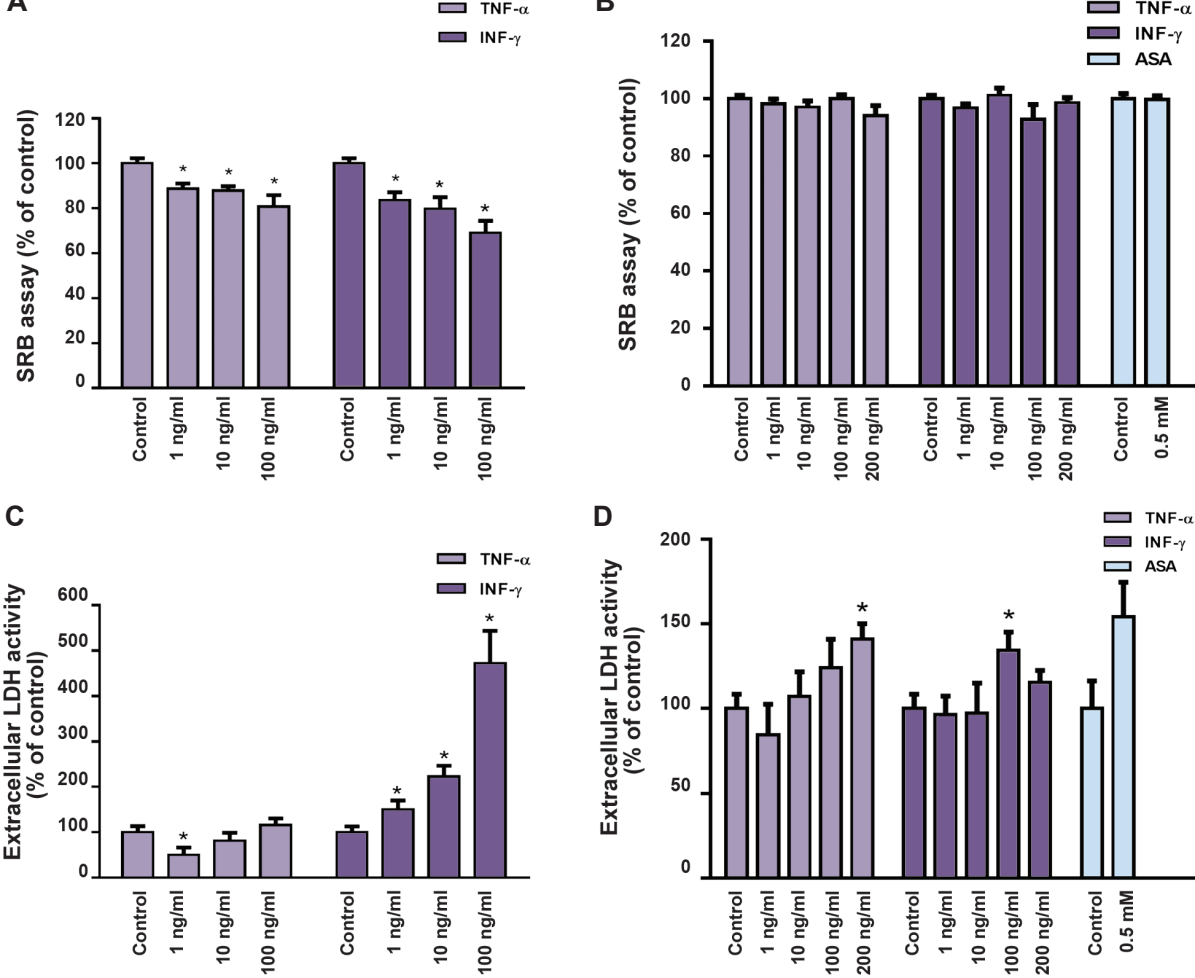
Effect of TNF-α (n=6-15), INF-γ (n=6-19) and ASA (n=14-20) (24 hours) on cell proliferation (A. IEC-6, B. Caco-2 cells) and viability (C. IEC-6 and D. Caco-2 cells) of IEC-6 and Caco-2 cells. Results are presented as arithmetic means ± SEM. TNF-α; TNF-tumour necrosis factor-alpha, INF-γ; Interferon-γ, ASA; Acetylsalicylic acid, SRB; Sulforhodamine B, LDH; Lactate dehydrogenase, and *; Significantly different from control (P<0.05).

In a first series of experiments, the effect of TNF-α and INF-γ upon the uptake of a low concentration of the short-chain fatty acid BT was tested in two different intestinal epithelial cell lines, the IEC-6 and the Caco-2 cell lines. In both cell lines, TNF-α and INF-γ decreased uptake of ^14^C-BT (Fig.1). The effect of both cytokines was more pronounced in IEC-6 cells. However, in this cell line, both cytokines presented an anti-proliferative and cytotoxic effect which can probably explain the decrease in ^14^C-BT uptake. In contrast, no anti-proliferative effect and only a slight cytotoxic effect of TNF-α and INF-γ were observed in Caco-2. So, inhibition of ^14^C-BT uptake in this cell line is most probably not related with cell loss (Fig.2). The effect of ASA on the uptake of a low concentration of ^14^C-BT was also tested on Caco-2 cells. We verified that ASA caused a very marked increase in the uptake of a low concentration of ^14^C-BT (Fig.1). This effect is not related to a change in cell proliferation or viability (Fig.2). 

### Influence of INF-γ and ASA upon the effect of BT upon cell proliferation, viability and differentiation

**Fig.3:**
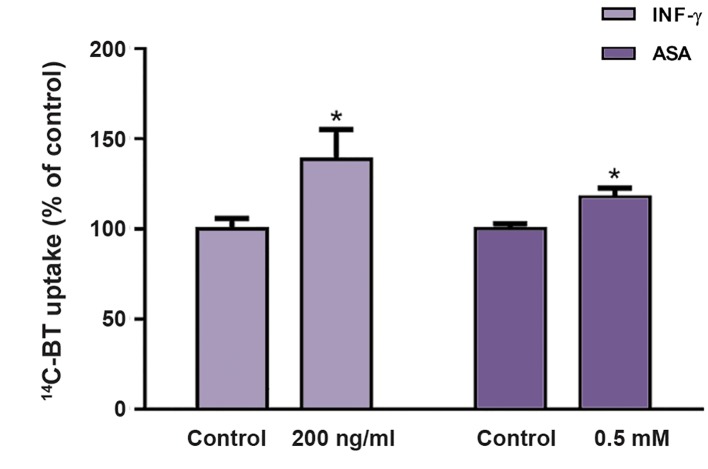
Effect of INF-γ (n=16) and ASA (n=20) (24 hours) on the uptake of a high concentration (10 mM) of ^14^C-BT by Caco-2 cells. Results are presented as arithmetic means ± SEM. INF-γ; Interferon-γ, ASA; Acetylsalicylic acid, BT; Butyrate, and *; Significantly different from control (P<0.05).

**Fig.4:**
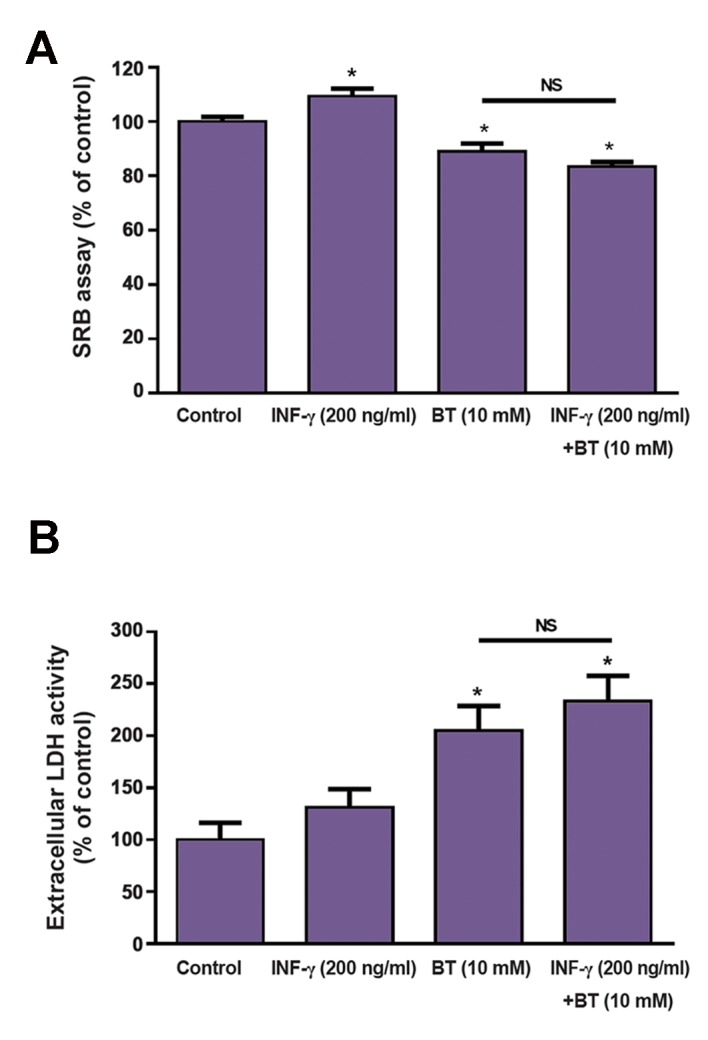
Influence of INF-γ 200 ng/ml (24 hours, n=16) upon the effect of BT (10 mM) on A. Proliferation and B. Viability of Caco-2 cells. Results are presented as arithmetic means ± SEM. INF-γ; Interferon-γ, BT; Butyrate, SRB; Sulforhodamine B, LDH; Lactate dehydrogenase, *; Significantly different from control (P<0.05), and NS; Not significantly different.

**Fig.5:**
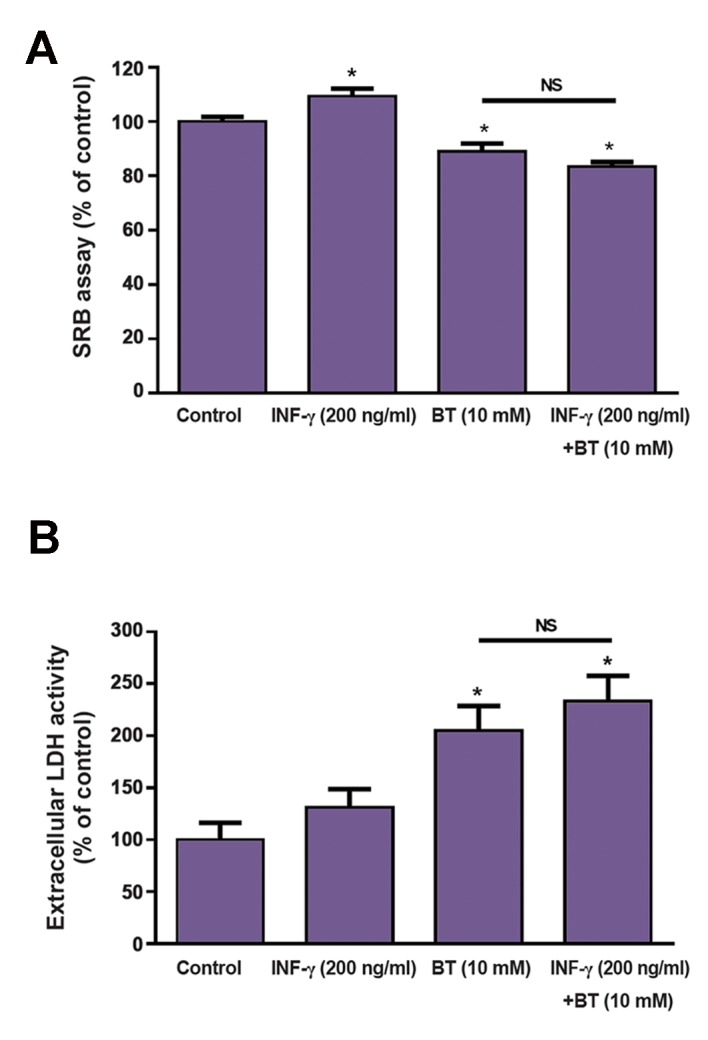
Influence of ASA 0.5 mM (24 hours, n=20) upon the effect of BT (10 mM) on A. Proliferation, B. Viability, and C. Differentiation of Caco-2 cells. Results are presented as arithmetic means ± SEM. ASA; Acetylsalicylic acid, BT; Butyrate, SRB; Sulforhodamine B, LDH; Lactate dehydrogenase, ALP; Alkaline phosphatase, *; Significantly different from control (P<0.05), **; Significantly different (P<0.05), and NS; Not significantly different.

The short-chain fatty acid BT possesses an anticarcinogenic effect at the intestinal level (4). Because, in Caco-2 cells, INF-γ and ASA caused significant changes in the uptake of ^14^C-BT not related to cell proliferation/viability changes, we decided to test the ability of these compounds to change the anticarcinogenic effect of BT. To this, Caco-2 cells (a tumoral cell line) were exposed to a high concentration of BT (10 mM) for 24 hours. First, we tested the efficacy of these two compounds in changing the uptake of a high concentration of 14 C-BT. Interestingly enough, both INF-γ and ASA caused a significantly increase in the uptake of a high concentration of ^14^C-BT (Fig.3). A high concentration of BT (10 mM, 24 hours) causes a very marked antiproliferative and cytotoxic effect in this cell line (Fig.4,5). The antiproliferative and cytotoxic effect of BT were not affected by the presence of IFN-γ (Fig.4); in contrast, the antiproliferative effect of BT was potentiated in the presence of ASA (Fig.5). 

### Effect of proinflammatory cytokines on ^3^H-FA uptake by Caco-2 cells 

**Fig.6:**
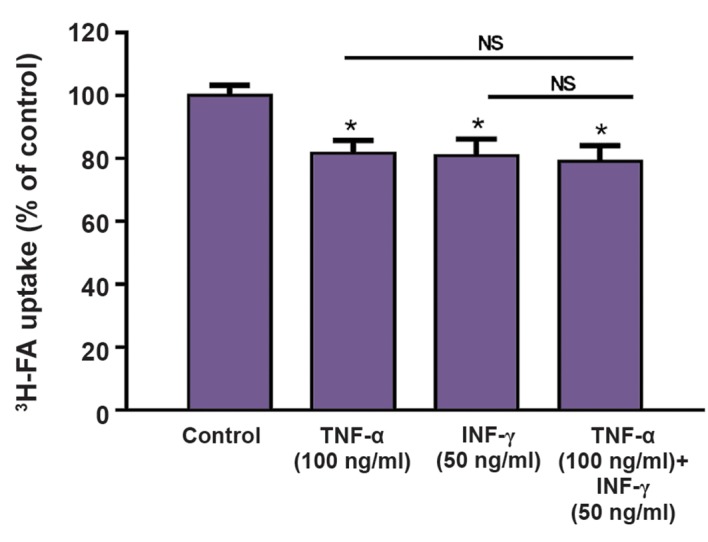
Effect of TNF-α (n=14), INF-γ (n=14) and TNF-α+INF-γ (n=12) (24 hours)on the uptake of ^3^H-FA 10 nM by Caco-2 cells. Results are presented as arithmetic means ± SEM. TNF-α; TNF-tumour necrosis factor-alpha, INF-γ; Interferon-γ, *; Significantly different from control (P<0.05), and NS, Not significantly different.

**Fig.7:**
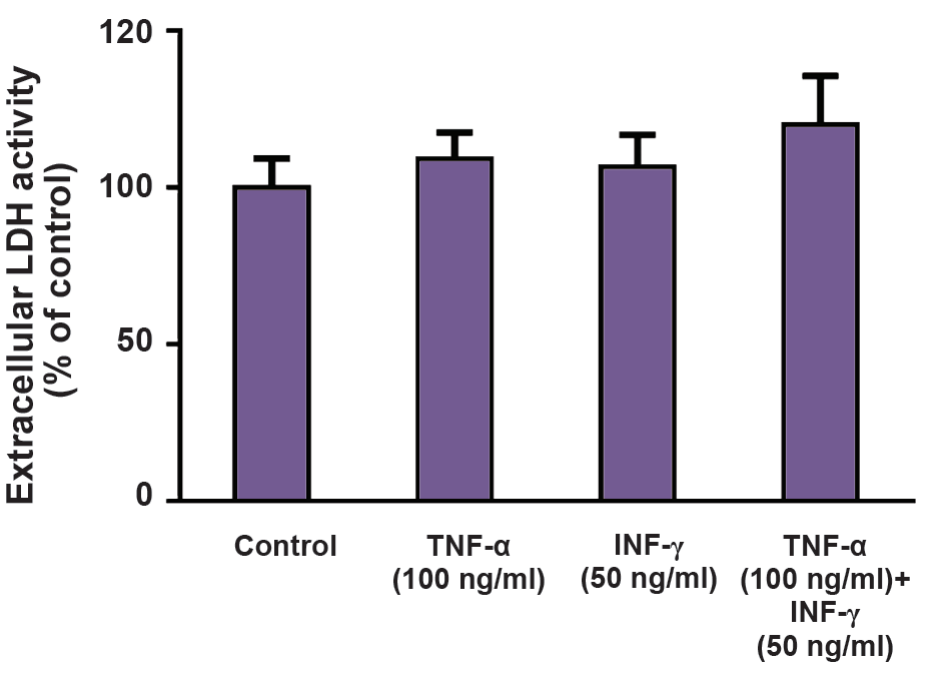
Effect of TNF-α (n=14), INF-γ (n=14) and TNF-α+INF-γ (n=12) (24 hours) on the viability of Caco-2 cells. Results are presented as arithmetic means ± SEM. TNF-α; TNF-tumour necrosis factor-alpha, INF-γ; Interferon-γ, LDH; Lactate dehydrogenase, and *; Significantly different from control (P<0.05).

We also tested the ability of TNF-α and INF- γin modulating the cellular uptake of ^3^H-FA by Caco-2 cells. Both cytokines were able to cause a significant decrease in the uptake of ^3^H-FA (Fig.6), without interfering with cell viability (Fig.7). However, combination of both cytokines did not result in a higher decrease in uptake (Fig.6). 

## Discussion

The aim of this work was to investigate the relationship between inflammatory status and absorption of nutrients with a role in CRC pathogenesis, more specifically the short-chain fatty acid BT and the vitamin folic acid. BT, produced in the colon by fermentation of dietary fiber, is the main energy source for colonocytes and promotes growth and proliferation of normal colonic epithelial cells (1). BT possesses also a protective role in the prevention and progression of colon carcinogenesis (4). This apparent opposing effect of BT upon growth of normal versus tumoral colonocytes has been referred to as the "BT paradox" (1,18). At the intestinal epithelial level, cellular uptake of BT involves two distinct transporters, the monocarboxylate transporter 1 (MCT1) (19) and the sodium- coupled moncarboxylate transporter 1 (SMCT1) (20). Interestingly enough, MCT1 expression is downregulated in the inflamed mucosa of IBD patients, in animal models of inflammation, and in response to the proinflammatory Th1 citokines TNF-α and IFN-γ (21). Similarly, SMCT1 expression is downregulated by TNF-α and IFN-γ (22,23). This reduction in both MCT1 and SMCT1- mediated BT uptake is probably responsible for the BT oxidation deficiency observed in intestinal inflammation. Indeed, several studies have found that BT oxidation is decreased in the inflamed mucosa of patients suffering from ulcerative colitis (24) or Crohn’s disease (25) and in animal models of experimental colitis (26). 

Interestingly enough, the proinflammatory citokines TNF-α and INF-γ were previously found to inhibit MCT1-mediated BT cellular uptake (21). Moreover, TNF-α and INF-γ were shown to decrease SMCT1-mediated BT cellular uptake (22,23). However, these previous studies did not compare the effect of TNF-α and INF-γ in normal and tumoral intestinal epithelial cell lines. Given the distinct cellular effects of BT in these two cell types, we decided to compare the effect of TNF-α and INF-γ in normal (IEC-6) and tumoral (Caco-2) intestinal epithelial cells. We verified that TNF-α and INF-γ were able to decrease ^14^C-BT uptake by both cell lines. Both compounds were found to be more potent in inhibiting ^14^C-BT uptake by non-tumoral IEC-6 cells when compared with tumoral Caco-2 cells. However, they presented a marked anti-proliferative and cytotoxic effect on IEC-6 cells (more evident for INF-γ than for TNF-α). In contrast, the inhibitory effect of the highest concentration of INF-γ tested (200 ng/ ml) upon ^14^C-BT uptake by Caco-2 cells was not associated with either an antiproliferative or a cytotoxic effect; TNF-α 200 ng/ml, although not showing an antiproliferative effect, caused a significant Caco-2 cell death. IBD patients possess a reduced number of BT-producing bacteria and consequently lower BT fecal concentration (27). Our results thus suggest that proinflammatory Th1 cytokines inhibit uptake of a low concentration of BT (10 µM), as found in IBD. Simultaneously, we show that these proinflammatory Th1 cytokines possess an antiproliferative and cytotoxic effect in normal intestinal epithelial cells, which suggests that they can induce alterations in intestinal barrier function *in vivo*. It remains unclear whether the loss of intestinal barrier function is a cause or a consequence of intestinal inflammation; however, intestinal epithelial cell injury with disruption of the intestinal barrier function is involved in IBD pathophysiology (28). Interestingly enough, BT promotes intestinal barrier function due to its ability to induce intestinal epithelial cell proliferation, differentiation and modulation of the expression of tight junction proteins (7). So, the antiproliferative and cytotoxic effect of the cytokines may not be the cause, but rather a consequence of inhibition of the cellular uptake of BT. 

In the next series of experiments, aimed at investigating the effect of proinflammatory cytokines upon the cellular effects of BT, we decided to test the effect of INF-γ (200 ng/ ml) upon the anticarcinogenic effect of BT in tumoral (Caco-2) cells. Previous works showed that high concentrations of BT (in the mM range) possess an antiproliferative and cytotoxic effect in Caco-2 cells (29,30). So, we investigated the ability of INF-γ to interfere with the uptake of a high concentration of BT (10 mM) and with its cellular effects. This concentration of BT (10 mM) is well within the physiological range of concentrations of this compound in the human colon (11-25 mM) under normal health conditions (31). In contrast to what was verified with a low concentration of ^14^C-BT (as found in IBD), INF-γ (200 ng/ml) caused a significant increase in the uptake of a high concentration of ^14^C-BT (10 mM) in Caco-2 cells. MCT1 is a low-affinity, high capacity BT transporter, while SMCT1 is a high-affinity, low activity BT transporter (19,20). We thus suggest that INF-γ inhibits SMCT1- mediated (predominant at low BT concentration) and increases MCT1-mediated and/or BT uptake mediated by passive diffusion (predominant at high BT concentration). One mechanism to explain the contribution of inflammation to carcinogenesis development is the formation of reactive species and induction of oxidative stress. Interestingly enough, we previously described that oxidative stress strongly inhibits SMCT1-mediated, but not MCT1-mediated BT uptake, and increases uptake of BT by passive diffusion (32). We verified that INF-γ did not interfere with the antiproliferative and cytotoxic effect of BT in Caco-2 cells. One hypothesis to explain the lack of efficacy of INF-γ in potentiating the anticarcinogenic effect of BT is the possibility that the cellular effects of BT are antagonized by INF-γ. It is important to note that BT possesses an anti-inflammatory effect at the intestinal level (4). In this context, it was recently described that BT induces inflammasome activation and production of IL-18 (33) while IFN-γ inhibits inflammasome activation (34). 

Cyclooxygenase-2 (COX-2) overexpression plays an important role in the inflammation- carcinogenesis pathway of CRC (35) and it is well established that the regular use of NSAIDs exerts a protective effect against CRC development (14). The effect of NSAIDs upon MCT1 and SMCT1 was previously investigated in a few studies. MCT1 appears to be inhibited by NSAIDS (e.g. indomethacin and ketoprofen) (31,32,36), while SMCT1 is inhibited by some NSAIDS (e.g. ibuprofen, ketoprofen, fenoprofen and naproxen) (37) while stimulated by others (e.g. diclofenac, meclofenamate and sulindac) (38). So, we decided to investigate the effect of ASA upon uptake of a low and a high concentration of ^14^C-BT by Caco- 2 cells, and to determine if this compound would interfere with the anticarcinogenic effect of BT in these cells. Interestingly enough, exposure to ASA was found to increase uptake of both a low and a high concentration of ^14^C-BT, not related to a cytotoxic or antiproliferative effect. Previous work from our group already described a stimulatory effect of ASA upon uptake of a high concentration of ^14^C-BT; however, only a short-term effect of ASA was investigated (32). Importantly, the antiproliferative effect of BT was potentiated by ASA. This result clearly shows that ASA is able to potentiate the anticarcinogenic effect of BT in tumoral intestinal epithelial cells. This mechanism might well contribute to the protective effect of ASA in relation to CRC. 

Folate possesses a dual role in CRC development and progression: it appears to have a protective role against neoplasia in normal colorectal mucosa, but it may also enhance pre-existing adenomatous lesions (3). IBD patients present significantly lower blood levels of folate (39). This vitamin deficiency can induce hyperhomocysteinemia that may potentiate T cell activation and differentiation into a Th1 phenotype (40). At the intestinal epithelial level, cellular uptake of dietary folate depends primarily on the activity of proton-coupled folate transporter (PCFT); however, the reduced folate carrier 1 and the folate receptor alpha may also be involved (41). A previous work from our group showed that oxidative stress strongly inhibits FA uptake and decreases mRNA levels of PCFT and RFC (42) and proinflammatory cytokines can induce oxidative stress. So, we decided to investigate the effect of TNF-α and INF-γ upon 3 H-FA uptake by Caco-2 cells. We verified that TNF-α and INF-γ caused a 20% reduction in the uptake of ^3^H-FA by Caco-2 cells. The decrease in FA uptake may put these cells on a FA deficient state, which will favor tumor progression (3). Additionally, clinically relevant antifolates are transported by folate membrane transporters; e.g. methotrexate (MTX) is transported by RFC and pemetrexed is an excellent substrate for PCFT as well as for RFC. Interestingly enough, the loss of RFC transport is an important mechanism of MTX resistance (43). So, inflammatory cytokines may also interfere with the chemotherapeutic efficacy of these drugs by decreasing their cellular uptake. 

## Conclusion

The inflammatory status has an impact upon cellular uptake of BT and FA, two nutrients with a role in CRC pathogenesis. Moreover, the anti- inflammatory ASA, by increasing the cellular uptake of BT, potentiates its anticarcinogenic effect in Caco-2 cells. 

## References

[1] Gonçalves P, Martel F (2016). Regulation of colonic epithelial butyrate transport: focus on colorectal cancer. Porto Biomed J.

[2] Aran V, Victorino AP, Thuler LC, Ferreira CG (2016). Colorectal cancer: epidemiology, disease mechanisms and interventions to reduce onset and mortality. Clin Colorectal Cancer.

[3] Cappellani A, Zanghì A, Di Vita M, Cavallaro A, Piccolo G, Veroux P (2013). Strong correlation between diet and development of colorectal cancer. Front Biosci (Landmark Ed).

[4] Bultman SJ (2017). Interplay between diet, gut microbiota, epigenetic events, and colorectal cancer.Mol Nutr Food Res.

[5] Davie JR (2004). Inhibition of histone deacetylase activity by butyrate. J Nutr.

[6] Marks P, Rifkind RA, Richon VM, Breslow R, Miller T, Kelly WK (2001). Histone deacetylases and cancer: causes and therapies. Nat Rev Cancer.

[7] Gonçalves P, Martel F (2013). Butyrate and colorectal cancer: the role of butyrate transport. Curr Drug Metab.

[8] Du W, Li WY, Lu R, Fang JY (2010). Folate and fiber in the prevention of colorectal cancer: between shadows and the light. World J Gastroenterol.

[9] Lucock M (2000). Folic acid: nutritional biochemistry, molecular biology and role in disease processes. Mol Genet Metab.

[10] Ames BN (2001). DNA damage from micronutrient deficiencies is likely to be a major cause of cancer. Mutat Res.

[11] Kim YI (2007). Folate and colorectal cancer: an evidence based critical review. Mol Nutr Food Res.

[12] Herszényi L, Barabás L, Miheller P, Tulassay Z (2015). Colorectal cancer in patients with inflammatory bowel disease: the true impact of the risk. Dig Dis.

[13] Abraham C, Cho JH (2009). Inflammatory bowel disease. N Engl J Med.

[14] Wakeman C, Keenan J, Eteuati J, Hollington P, Eglinton T, Frizelle F (2015). Chemoprevention of colorectal neoplasia. ANZ J Surg.

[15] Bergmeyer HU, Bernt E, Bergmeyer HU (1974). Lactate dehydrogenase. Methods in enzymatic analysis.

[16] Araújo JR, Correia-Branco A, Ramalho C, Keating E, Martel F (2013). Gestational diabetes mellitus decreases placental uptake of long-chain polyunsaturated fatty acids: involvement of long-chain acyl-CoA synthetase. J Nutr Biochem.

[17] Bradford MM (1976). A rapid and sensitive method for the quantitation of microgram quantities of protein utilizing the principle of protein-dye binding. Anal Biochem.

[18] Burgess DJ (2012). Metabolism: warburg behind the butyrate paradox?. Nat Rev Cancer.

[19] Halestrap AP (2013). Monocarboxylic acid transport. Compr Physiol.

[20] Gupta N, Martin PM, Prasad PD, Ganapathy V (2006). SLC5A8 (SMCT1)-mediated transport of butyrate forms the basis for the tumor suppressive function of the transporter. Life Sci.

[21] Thibault R, De Coppet P, Daly K, Bourreille A, Cuff M, Bonnet C (2007). Down-regulation of the monocarboxylate transporter 1 is involved in butyrate deficiency during intestinal inflammation. Gastroenterology.

[22] Borthakur A, Anbazhagan AN, Kumar A, Raheja G, Singh V, Ramaswamy K (2010). The probiotic Lactobacillus plantarum counteracts TNF-{alpha}-induced downregulation of SMCT1 expression and function. Am J Physiol Gastrointest Liver Physiol.

[23] Mathewson ND, Jenq R, Mathew AV, Koenigsknecht M, Hanash A, Toubai T (2016). Gut microbiome-derived metabolites modulate intestinal epithelial cell damage and mitigate graft-versus-host disease. Nat Immunol.

[24] Den Hond E, Hiele M, Evenepoel P, Peeters M, Ghoos Y, Rutgeerts P (1998). In vivo butyrate metabolism and colonic permeability in extensive ulcerative colitis. Gastroenterology.

[25] Duffy MM, Regan MC, Ravichandran P, O'Keane C, Harrington MG, Fitzpatrick JM (1998). Mucosal metabolism in ulcerative colitis and Crohn’s disease. Dis Colon Rectum.

[26] Ahmad MS, Krishnan S, Ramakrishna BS, Mathan M, Pulimood AB, Murthy SN (2000). Butyrate and glucose metabolism by colonocytes in experimental colitis in mice. Gut.

[27] Sokol H, Seksik P, Furet JP, Firmesse O, Nion-Larmurier I, Beaugerie L (2009). Low counts of Faecalibacterium prausnitzii in colitis microbiota. Inflamm Bowel Dis.

[28] Salim SY, Söderholm JD (2011). Importance of disrupted intestinal barrier in inflammatory bowel diseases. Inflamm Bowel Dis.

[29] Gonçalves P, Araújo JR, Martel F (2011). Characterization of butyrate uptake by nontransformed intestinal epithelial cell lines. J Membr Biol.

[30] Gonçalves P, Araújo JR, Pinho MJ, Martel F (2009). Modulation of butyrate transport in Caco-2 cells. Naunyn Schmiedebergs Arch Pharmacol.

[31] Hallert C, Björck I, Nyman M, Pousette A, Grännö C, Svensson H (2003). Increasing fecal butyrate in ulcerative colitis patients by diet: controlled pilot study. Inflamm Bowel Dis.

[32] Gonçalves P, Gregório I, Catarino TA, Martel F (2013). The effect of oxidative stress upon the intestinal epithelial uptake of butyrate. Eur J Pharmacol.

[33] Macia L, Tan J, Vieira AT, Leach K, Stanley D, Luong S (2015). Metabolite-sensing receptors GPR43 and GPR109A facilitate dietary fibre-induced gut homeostasis through regulation of the inflammasome. Nat Commun.

[34] Mishra BB, Rathinam VA, Martens GW, Martinot AJ, Kornfeld H, Fitzgerald KA (2013). Nitric oxide controls the immunopathology of tuberculosis by inhibiting NLRP3 inflammasome-dependent processing of IL-1β. Nat Immunol.

[35] Tuncer S, Banerjee S (2015). Eicosanoid pathway in colorectal cancer: Recent updates. World J Gastroenterol.

[36] Choi JS, Jin MJ, Han HK (2005). Role of monocarboxylic acid transporters in the cellular uptake of NSAIDs. J Pharm Pharmacol.

[37] Itagaki S, Gopal E, Zhuang LN, Fei YJ, Miyauchi S, Prasad PD (2006). Interaction of ibuprofen and other structurally related NSAIDs with the sodium-coupled monocarboxylate transporter SMCT1 (SLC5A8). Pharm Res.

[38] Ananth S, Zhuang L, Gopal E, Itagaki S, Ellappan B, Smith SB (2010). Diclofenac-induced stimulation of SMCT1 (SLC5A8) in a heterologous expression system: a RPE specific phenomenon. Biochem Biophys Res Commun.

[39] Erzin Y, Uzun H, Celik AF, Aydin S, Dirican A, Uzunismail H (2008). Hyperhomocysteinemia in inflammatory bowel disease patients without past intestinal resections: correlations with cobalamin, pyridoxine, folate concentrations, acute phase reactants, disease activity, and prior thromboembolic complications. J Clin Gastroenterol.

[40] Dawson H, Collins G, Pyle R, Deep-Dixit V, Taub DD (2004). The immunoregulatory effects of homocysteine and its intermediates on T-lymphocyte function. Mech Ageing Dev.

[41] Zhao R, Matherly LH, Goldman ID (2009). Membrane transporters and folate homeostasiss: intestinal absorption and transport into systemic compartments and tissues. Expert Rev Mol Med.

[42] Couto MR, Gonçalves P, Catarino T, Araújo JR, Correia-Branco A, Martel F (2012). The effect of oxidative stress upon the intestinal uptake of folic acid: in vitro studies with Caco-2 cells. Cell Biol Toxicol.

[43] Hou Z, Matherly LH (2014). Biology of the major facilitative folate transporters SLC19A1 and SLC46A1. Curr Top Membr.

